# Preclinical evaluation of an unconventional ruthenium‐gold‐based chemotherapeutic: RANCE‐1, in clear cell renal cell carcinoma

**DOI:** 10.1002/cam4.2322

**Published:** 2019-06-13

**Authors:** Benelita T. Elie, Karen Hubbard, Yuriy Pechenyy, Buddhadev Layek, Swayam Prabha, Maria Contel

**Affiliations:** ^1^ Department of Chemistry Brooklyn College, The City University of New York Brooklyn New York; ^2^ Biology PhD Program, The Graduate Center The City University of New York New York New York; ^3^ Department of Biology City College of New York, The City University of New York New York New York; ^4^ University of Minnesota College of Pharmacy Minneapolis Minnesota; ^5^ Chemistry PhD Program, The Graduate Center The City University of New York New York New York; ^6^ Biochemistry PhD Program, The Graduate Center The City University of New York New York New York

**Keywords:** clear cell renal cell carcinoma, histopathology, kidney metastasis, mice xenograft model, pharmacokinetics, unconventional chemotherapeutics

## Abstract

**Background:**

There are few effective treatments for patients with advanced clear cell renal cell carcinoma (CCRCC). Recent findings indicate that ruthenium‐gold containing compounds exhibit significant antitumor efficacy against CCRCC in vitro affecting cell viability as well as angiogenesis and markers driving those 2 phenomena. However, no in vivo preclinical evaluation of this class of compounds has been reported.

**Methods:**

Following the dose‐finding pharmacokinetic determination, NOD.CB17‐Prkdc SCID/J mice bearing xenograft CCRCC Caki‐1 tumors were treated in an intervention trial for 21 days at 10 mg/kg/72h of RANCE‐1. At the end of the trial, tumor samples were analyzed for histopathological and changes in protein expression levels were assessed.

**Results:**

After 21 days of treatment there was no significant change in tumor size in the RANCE‐1‐treated mice as compared to the starting size (+3.87%) (*P* = 0.082) while the vehicle treated mice exhibited a significant tumor size increase (+138%) (*P* < 0.01). There were no signs of pathological complications as a result of treatment. Significant reduction in the expression of VEGF, PDGF, FGF, EGFR, and HGRF, all key to the proliferation of tumor cells and stromal cells serving protumorigenic purposes was observed.

**Conclusions:**

The tumor growth inhibition displayed and favorable pathology profile of RANCE‐1 makes it a promising candidate for further evaluation toward clinical use for the treatment of advanced CCRCC.

## 1 INTRODUCTION

**Chart 1:**
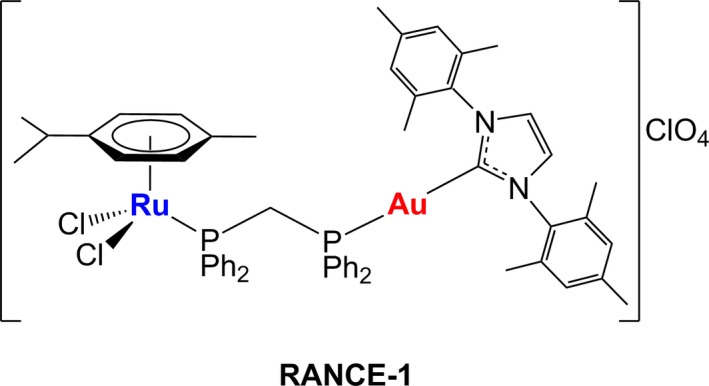
Compound used in this study: bimetallic [Cl_2_(p‐cymene)Ru(μ‐dppm)Au(IMes)]ClO_4_ (RANCE‐1).^20^, ^21^

In adults, renal cell carcinoma (RCC) constitutes the most common type of kidney cancer (85%).^1^ In this population ca 70% of the patients present a disease that fits the histological parameters of clear cell renal cell carcinoma (CCRCC).^2^ There were 400 000 new cases of kidney cancer worldwide and in about 30% of new diagnoses, the cancer will have already mestastasized.^3^ Kidney cancer affects both men and women in a ratio 2:1 and is diagnosed largely in individuals over 64 years of age or with known risk factors (including age, smoking, obesity, hypertension, treatment for kidney failure, inherited genetic syndromes, family history, specific ethnicity, occupational exposure, and chronic misuse of over the counter pain relief drugs). There are no efficient treatment options for advanced stage and metastatic CCRCC and therefore the rates of 5‐year disease‐free survival is about 12% when the cancer has spread beyond the kidney capsule and into surrounding and distal tissues. For advanced stages of CCRCC, pharmacological interventions such as chemotherapy, targeted therapy or immunotherapy are the preferred options. These options are however limited and 5‐FU and capecitabine are the most effective chemo‐monotherapies (rates of ca 20% response rate and survival of 15 months).^4^ Immunotherapy treatments such as IL‐2 and INF‐α, combination of immunomodulators nivolumab and ipilimumab, or the use of bevacizumab (VEGF‐A inhibitor) have also afforded modest responses (9‐20 months survival rate).^5^ Targeted therapies such as sunitinib, pazopanib, or temsirolimus are the “standard of care” for first‐line treatment.^6^ Combination of chemotherapy with targeted therapies have shown promising results in clinical trials but also within the range of 20 months of survival.^7^ Therefore, there is a need to develop new therapies for CCRCC to be used either alone or in combination treatments to improve therapeutic outcomes. Metal‐based chemotherapeutics such as platinum‐based drugs in combination or monotherapy regimes are used to treat a large number of cancers but their efficacy is still hindered by clinical problems, including acquired or intrinsic resistance, a limited spectrum of activity, and high toxicity leading to significant side effects.^8^ A number of unconventional metal‐based compounds highly effective in cancers resistant to cisplatin and other chemotherapeutic agents but with fewer side effects*,* have been described over the past decade (including recent successful clinical trials).^9^, ^10^, ^11^ Our laboratory has developed drugs containing 2 different active metal‐based fragments in the same molecule (heterometallic) to enhance the anticancer properties of single metallodrugs. The hypothesis is that the incorporation of 2 different biologically active metals in the same molecule may improve their antitumor activity as a result of metal specific interactions with distinct biological targets (cooperative effect) or by the improved physicochemical properties of the resulting heterometallic compound (synergism).^12^ We have focused on gold (Au)‐based compounds containing a second metal (titanium or ruthenium) (Chart 1). We have shown that specific titanium‐gold based derivatives have high efficacy against ovarian and prostate cancers in vitro^13^, ^14^ and renal cancer both in vitro^15^, ^16^, ^17^ and in vivo.^18^ We also reported on ruthenium (Ru)‐Au based complexes with in vitro efficacy against HCT 116 colon cancer cell lines^19^ and most recently in vitro against CCRCC.^20^, ^21^

We report here on the high efficacy in vivo (subcutaneous CCRCC Caki‐1 xenograft mice model) of a selected bimetallic Ru‐gold (Au) compound, RANCE‐1 (structure in Chart 1). We have detailed here the results of the in vivo efficacy trial, pharmacokinetic and histopathological studies as well as preliminary mechanistic studies.

## 2 MATERIALS AND METHODS

### 2.1 Cells

Caki‐1, a human epithelial CCRCC cell line derived from a metastasis to the skin was newly obtained for these studies from the American Type Culture Collection (ATCC) (Manassas, VA) and cultured in Roswell Park Memorial Institute (RPMI‐1640) (Mediatech Inc, Manassas, VA) media containing 10% foetal bovine serum (FBS, Life Technologies, Grand Island, NY), 1% Minimum Essential Media (MEM) nonessential amino acids (NEAA, Mediatech) and 1% penicillin‐streptomycin (PenStrep, Mediatech) and incubated at 37°C and 5% CO2 in a humidified incubator.

### 2.2 Determination of maximum tolerated dose of RANCE‐1

Maximum tolerated dose (MTD) of RANCE‐1 in naïve NOD.CB17‐Prkdc SCID/J mice. Following 6 intraperitoneal (ip) doses between 30 mg/kg/48 h and 50 mg/kg/48 h followed by a 2‐week recovery period. Vehicle solution (0.5% DMSO + 99.5% normal saline) treated mice were used as controls. Lung, liver, kidney, spleen, and heart were collected, weighed and visually evaluated during a gross necropsy. Parameters such as physical distress and mortality were monitored.

### 2.3 In vivo biodistribution analysis of RANCE‐1

Female and male NOD.CB17‐Prkdc scid/J mice bearing subcutaneous (subcu) Caki‐1 tumors and treated with RANCE‐1 (10 mg/kg, ip) were used for pharmacokinetic and biodistribution studies. Blood was collected from submandibular vein using a heparin coated glass capillary into heparinized blood collection tubes on ice at time intervals of 1, 2, 6, 12, 24, 48, and 72 hours post injection. Plasma was harvested by centrifuging blood samples at 2800 rpm for 15 minutes at 4°C and stored frozen at − 80°C until analysis. Similarly, kidney, liver, and tumor were harvested after final time point, weighed, and stored into glass vials. One mL of deionized water was added to each tissue sample, subjected to ultrasonic homogenization at 15 W power for 1 minute, followed by lyophilization.

Plasma and tissue concentrations of Ru and Au were measured using inductively coupled plasma–mass spectrometry (ICP‐MS). Plasma (10 μL) or tissue samples were transferred into glass vials, and 1 mL of a 75:25 mixture of nitric acid (16 N) and hydrochloric acid (12 N) was added to each vial. The mixture was then heated at 90°C for 5 hours. After cooling to room temperature, the samples were centrifuged to remove debris if any. All samples were then mixed with 40 ppb of indium internal standard and analyzed using a Thermo Scientific XSERIES 2 ICP‐MS coupled with ESI PC3 Peltier cooled spray chamber, SC‐FAST injection loop, and SC‐4 autosampler. All the elements were measured using He/H_2_ collision‐reaction mode. Plasma and tissue samples from control mice were spiked with known concentration of RANCE‐1 to determine its extraction efficiency.

### 2.4 Post intervention trial biodistribution

At the end of the intervention trial in which female and male NOD.CB17‐Prkdc scid/J mice bearing subcutaneous Caki‐1 tumors were treated with 10mg/kg/72h of RANCE‐1 ip over 21 days, liver, kidney, and tumor of the animals were harvested, weighed, and transferred into glass vials. Tissue samples were processed as described above and analyzed for Ru and Au content using ICP‐MS

Pharmacokinetic parameters were obtained from the plasma concentration–time profiles by noncompartmental analysis using Phoenix WinNonlin 6.4 version 6.4 (Pharsight Corporation, Mountain View, CA). The pharmacokinetic parameters quantified were the maximum plasma drug concentration (C_max_), the time to reach C_max_ (T_max_), the area under the plasma concentration–time curve from 0 hour to last measurable concentration (AUC_last_), elimination rate constant (ke), plasma half‐life (t_1/2_), apparent total clearance of the drug from plasma (Cl/F), and apparent volume of distribution (Vd/F). Concentrations of ruthenium and gold in liver, kidney, and tumors were also determined.

### 2.5 Preparation of histological samples and immunohistochemistry

Tissue sections were prepared by embedding in OCT (Thermo Fisher Scientific, Waltham, MA) followed by freezing at − 80°C. Vessel leakiness was evaluated following tail‐vein injection of 100 μL of Dextran Texas Red (Invitrogen). Vessel integrity was assessed after tail‐vein injection of 50 μL of lectin labelled with FITC (Vector Labs, Burlingame, CA). Apoptotic cells were visualized using an anti‐rabbit cleaved caspase 3 primary antibody (Cell Signaling Technology, Danvers, MA) and an goat‐anti‐rabbit Alexa Fluor 594 secondary antibody (Cell Signaling Technology); proliferating cells were visualized using an anti‐mouse Ki67 primary antibody (Cell Signaling Technology) and an goat‐anti‐mouse Alexa Fluor 488 secondary antibody (Cell Signaling Technology); DAPI containing ProLong Gold Antifade Mounting Medium (Cell Signaling Technology) was used to visualize the nuclei and mount the slide.

### 2.6 Analysis of cell proliferation and apoptosis

For all histological analyses, tumors from treated mice were compared to those of the vehicle control of the intervention trial. Samples from 4 mice per treatment group were analyzed. Stained samples were imaged at 20× magnification (ZEISS LSM 700). Cell proliferation and apoptosis were quantified one channel at a time using ImageJ (NIH, open access software) and were calculated as the percentage of Ki67 positive or cleaved caspase 3 positive cells per DAPI positive cells per field of view.

### 2.7 Preparation of samples for pathology

At the end of the intervention trial, female and male NOD.CB17‐Prkdc scid/J mice bearing subcutaneous Caki‐1 tumors were euthanized, blood was collected by intracardiac puncture and the carcasses were perfused with 4% PFA. Lung, kidney, heart, spleen, lymphatic tissue were collected, mounted in paraffin, sectioned and stained by Hemotoxilyn & Eosin. Samples were imaged at 20× under light microscope for analysis.

### 2.8 Analysis of changes in protein expression

Whole tumors were extracted at the trial endpoint and lysed. Before application to the array, protein concentration was determined by BCA. Then 150 µg of lysate was incubated for 24 hours with the Proteome Profiler Human XL Oncology Array (ARY026, R&D Systems) and Human Cell Stress Array (ARY018, R&D Systems). The relative expression levels of the proteases were determined according to the manufacturer's protocol, and signal intensities were compared using HLImage^++^ software.

### 2.9 Data collection and statistical analysis

Results for all experiments were expressed as mean ± Standard Error of the Mean. Experiments were conducted in duplicate or triplicate. For all other parameters, statistical significance was determined using an independent 2‐tailed Student *t* test and one‐way ANOVA for independent data. *P* < 0.05 was considered as significant for all statistical analyses. All statistical analysis was done using GraphPad Prism® software (La Jolla, CA).

## 3 RESULTS

### 3.1 Toxicity studies

The MTD study indicated that male and female mice tolerated 3 ip injections at doses of 35 mg/kg/48 h of RANCE‐1 without notable signs of toxicity or changes in pathological parameters in the treated animals. There were, upon gross‐necropsy, no obvious signs of local toxicity were observed in the peritoneal cavity. This dose range and toxicity study demonstrates that RANCE‐1 is a relatively safe compound within a 2 week treatment at doses at or below 35 mg/kg ip These findings informed the rationale for selecting the doses of 10mg/kg (1/4 MTD) of RANCE‐1 for the subsequent pharmacokinetic (PK), intervention trials and pathology analyses.

### 3.2 Pharmacokinetics and biodistribution

**Figure 1:**
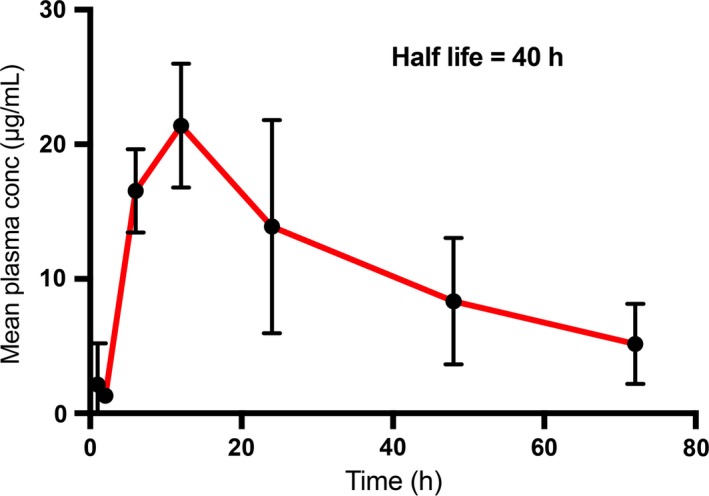
Plasma concentration of the Ru metal of RANCE‐1 at various intervals after single ip injection. The Ru metal concentration determination is performed by ICP‐MS (see details in the methods section). Data represent mean ± SD (n = 4)

**Figure 2:**
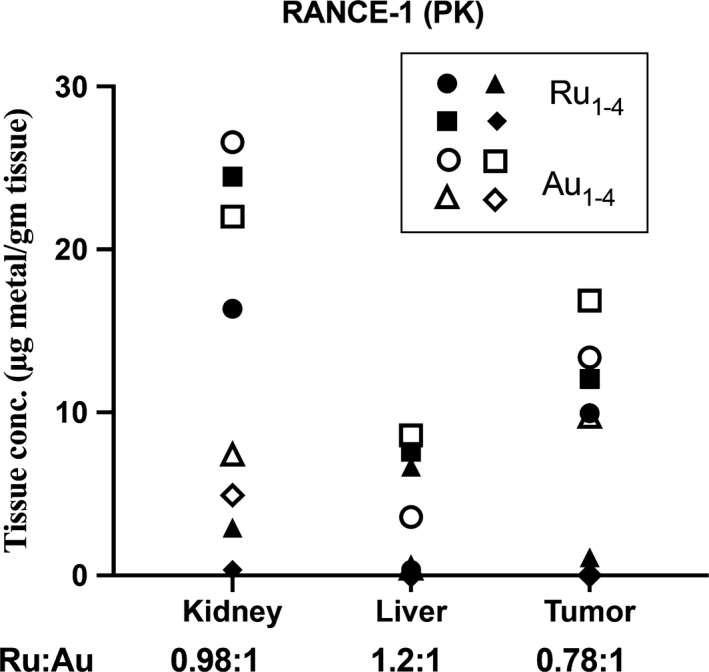
Comparison of Ru and Au tissue concentrations for each animal at 72 h post injection of RANCE‐1. Below the *x*‐axis, Ru:Au represents the ratio of the 2 metals in each tissue. The Ru and Au metal concentration determination was performed by ICP‐MS (see details in the methods section)

After the ip administration of a single dose of 10 mg/kg RANCE‐1 followed by time lapse blood collection and terminal tissue collection in xenograft Caki‐1 tumor bearing NOD.CB17‐Prkdc SCID/J mice, it was determined that the compound was absorbed slowly, as indicated by a peak plasma concentration of Ru reached at 10 h after dosing. RANCE‐1 eliminated (possibly in urine unchanged) slowly from the blood with an elimination half‐life (t_1/2_) of 40.8 hours (Table 1 and Figure 1). This informed the choice of a 72h window between doses, as this allowed sufficient time for clearance of the compound. There was a significant amount of both Ru and Au found in the tumor tissue (Figure 2). It is important to note that we observed close to a 1:1 ratio of both metals (Ru and Au) in the tissue.

**Table 1:** Pharmacokinetic parameters of RANCE‐1 after single ip injection in tumor‐bearing mice

Parameters	RANCE‐1
Dose	10 mg/kg, ip
T_max_ (h)	10.00 ± 3.46
C_max_ (µg/mL)	22.60 ± 2.58
AUC_0‐72h _(µg h/mL)	792.9 ± 270.3
ke (h^‐1^)	0.0196 ± 0.0088
t_1/2_ (h)	40.77 ± 18.67
V_d_/F (mL/Kg)	612.10 ± 429.00
Cl/F (mL/h/Kg)	10.00 ± 3.15
C_minss_ (µg/mL)^a^	7.17 ± 3.91
Time to reach steady state (h)	203.8 ± 93.4

Pharmacokinetic parameters determined include the maximum observed plasma concentration (C_max_), the time to reach C_max_ (T_max_), area under the plasma concentration–time curve from time zero to 72 h post dose (AUC_0‐72h_), elimination rate constant (ke), terminal elimination half‐life (t_1/2_), apparent total clearance from plasma (Cl/F), apparent volume of distribution (V_d_/F), and minimum plasma drug concentrations at steady state (C_minss_).

a The predicted C_minss_ is based on a dose of 10 mg/kg every 72 h.

### 3.3 Efficacy

**Figure 3:**
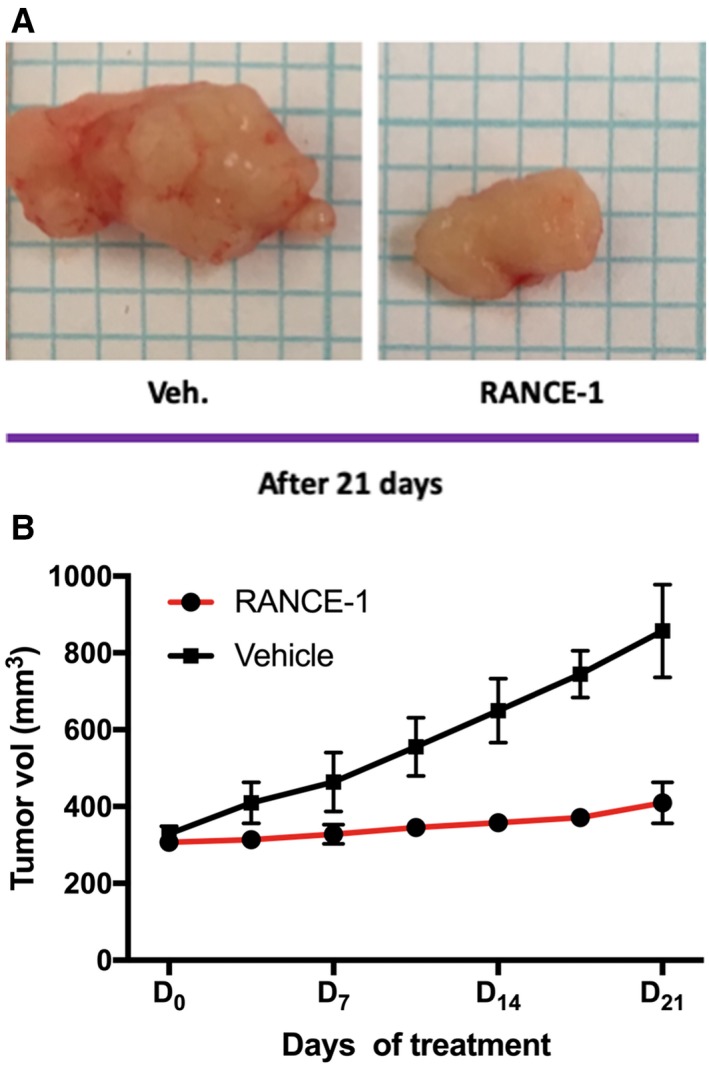
RANCE‐1 treatment inhibits tumor growth in a xenograft renal cancer intervention trial. Percent of change in tumor burden in a cohort of 3 females and 3 males NOD.CB17‐Prkdc scid/J mice inoculated subcutaneously with 5 × 10^6^ Caki‐1 cells. The treatment started when tumors were palpable (~5 mm diameter). Six mice were treated with RANCE‐1 (red line) administered in the amount of 10 mg/kg/72h i.p, 6 were treated with the vehicle 100 μL of the vehicle (0.5% DMSO + 99.5% normal saline) (black line) administered once/72h i.p for 21 days and tumor burden was measured by caliper twice a week. (A) Representative histological samples of tumors resected from each treatment group. (B) The average tumor volume after 21 days of treatment did not significantly change in RANCE‐1 treated mice, average tumor growth 3.87% (*P* = 0.082) compared to the vehicle (Veh) control‐treated group where tumor volume increased by 138%, ** *P* < 0.01. Mice were treated with RANCE‐1 (10 mg/kg/72h), or Veh by intraperitoneal injection from for 21 days. RANCE‐1: n = 6 mice, Veh: n = 6 mice

The in vivo anticancer efficacy of RANCE‐1 was determined in xenograft Caki‐1 tumor bearing NOD.CB17‐Prkdc SCID/J mice following ip administration of seven doses spaced by 72h followed by a 72h recovery period before sacrifice. In mice treated with RANCE‐1, we observed neither tumor growth nor shrinkage from the starting tumor burden recorded at the beginning of the trial. RANCE‐1 had a chemo‐static effect on tumor growth. Mice treated with RANCE‐1 had no change in tumor burden after 21 days of treatment (a total of 7 doses) (Figure 3). This result was promising given that few metallodrugs yield this degree of growth inhibition. We observed a growth in tumor volume of 138% in the vehicle treated mice and no significant change in weight or any decline in the well‐being of the RANCE‐1 treated mice were observed during this trial. These results indicate that RANCE‐1 has relevant growth inhibiting properties.

### 3.4 Mechanism of action

**Figure 4:**
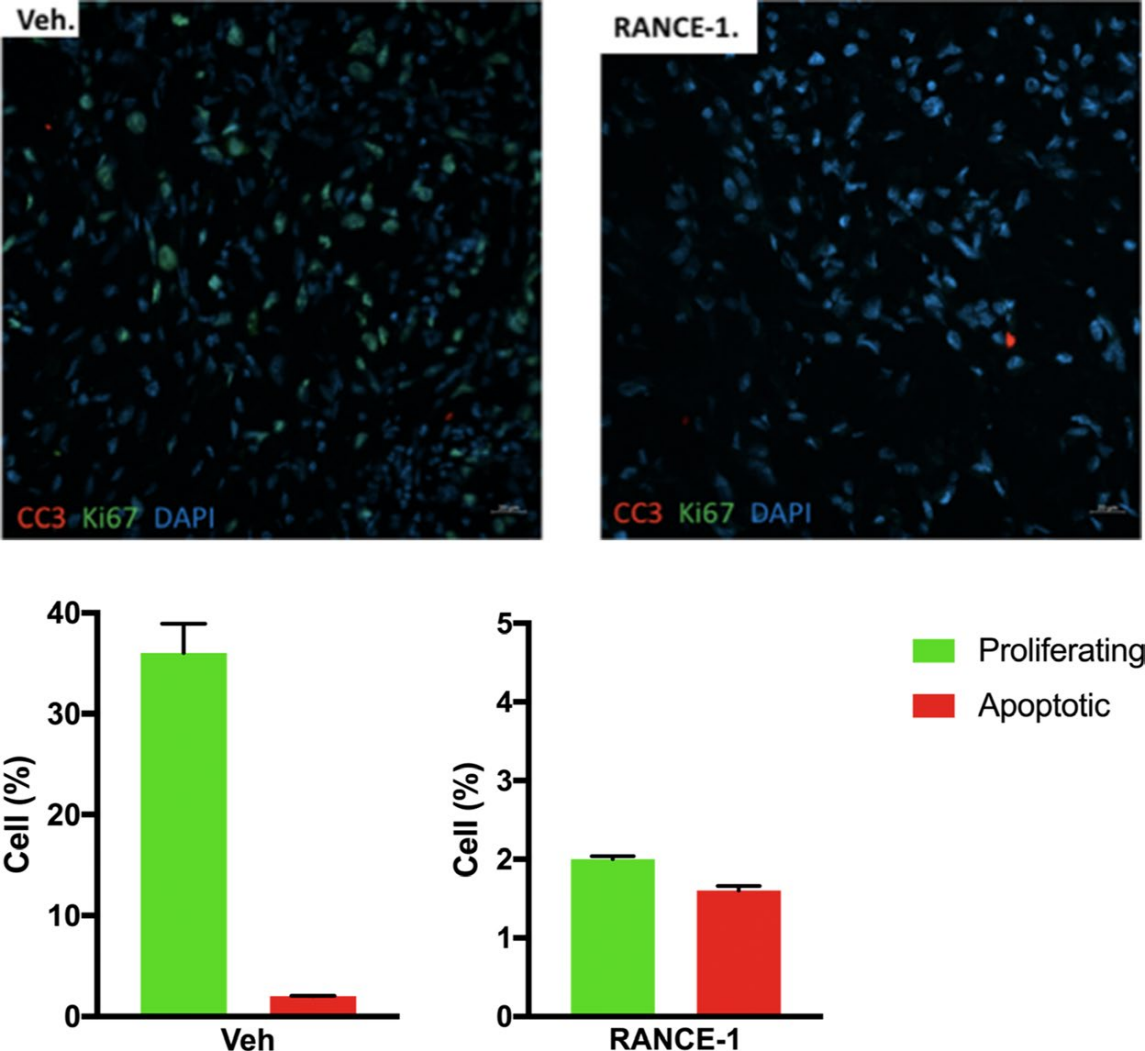
Effects of RANCE‐1 treatment on the balance between cell proliferation and apoptosis in Caki‐1 xenograft tumors. RANCE‐1 treatment affects the proliferation and apoptosis ratio/equilibrium at the 21‐day trial endpoint. (A) Proliferating cells were detected by Ki67 staining (green) and apoptotic cells were detected by cleaved caspase 3 staining (red). Representative images are shown for Vehicle‐treated tumors (left panel) and RANCE‐1 treated tumors (right panel) at the 21day trial endpoint. (B) The percentage of proliferating cells (Ki67 positive) and the percentage of apoptotic cells (cleaved caspase 3 positive) over the total number of DAPI‐positive cells in the tumor. RANCE‐1 treatment resulted in a 36% decrease in cell proliferation and a 0.4% decrease in apoptosis. (apoptosis RANCE‐1 *P* = 0.71, proliferation RANCE‐1 ***P < 0.001). Data represent mean ± SEM (n = 6 mice per group)

RANCE‐1 did affect the proliferation to apoptosis cell ratio in the xenograft model (Figure 4). Because tumor growth can also reflect changes in cell death, we compared the proliferation rate to apoptotic rates using the proliferation marker ki‐67 and apoptotic marker cleaved caspase 3. The average of proliferating cells in individual tumors of the vehicle (Veh) treated mice control group was 36%. RANCE‐1 reduces tumor cell proliferation by 18‐fold (from 36% for the Veh treated to 2% for the RANCE‐1 treated) and does not significantly affect apoptosis (from 2% for the Veh treated to 1.6% for the RANCE‐1 treated‐ mice). These findings are interesting because, the tumors did not seem to gain volume, as compared to vehicle treated mice, and this is reflected by the difference in proliferation to apoptosis ratio of the 2 treatment groups.

### 3.5 Protein expression analysis

**Figure 5:**
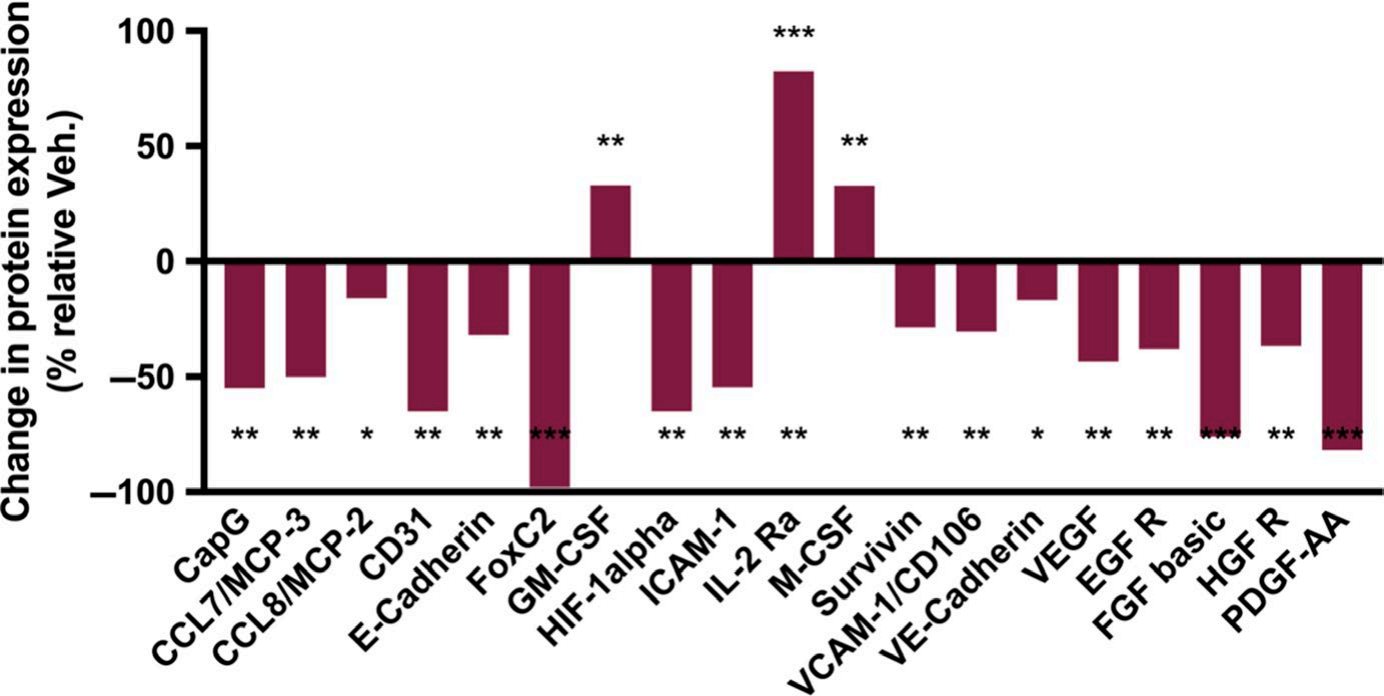
RANCE‐1 treatment induces tumor changes in expression growth factors and cancer‐immune interface regulators in vivo. In vivo changes in expression levels of proteins of oncological interest, following a 21‐day efficacy trial in Caki‐1 xenograft tumor bearing NOD.CB17‐Prkdc SCID/J mice. The values indicate the percentage of change in expression relative to DMSO treated cells in a Proteome Profiler immunoblotting assay. (*P < 0.05, **P < 0.01, ***P < 0.001). Data represent relative change from 2 separate samples for RANCE‐1 and Vehicle treated mice

**Figure 6:**
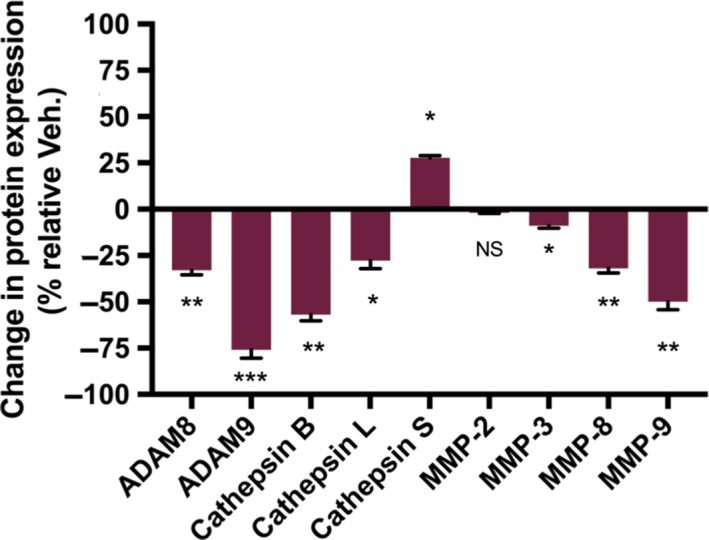
RANCE‐1 treatment induces changes in expression of proteolytic factors in vivo. In vivo changes in expression levels of pericellular proteolytic factors or activators of proteolytic factors, following a 21‐day efficacy trial in Caki‐1 xenograft tumor bearing NOD.CB17‐Prkdc SCID/J mice. Analysis of 150 µg of protein extracted from cell or tumor lysate using a Proteome Profiler immunoblotting assay. The values indicate the percentage of change in expression relative to DMSO treated cells. (NS = Not significant, *P < 0.05, **P < 0.01 ***P < 0.001). Data represent relative change from 2 separate samples for RANCE‐1 and Vehicle treated mice

Given the significant decrease in proliferation in tumor tissue, the effects of treatment with RANCE‐1 on xenograft tumors protein expression was explored. It was observed from the histological analysis (Figures 5 and 6) that in response to RANCE‐1 treatment, there was a striking reduction in proliferation, which could be mediated by changes in the expression of proliferative factors. There was not only a decrease in the expression of many growth factors including VEGF, PDGF, FGF, EGFR, and HGRF, all key to the proliferation of tumor cells, but also for stromal cells serving protumorigenic purposes. We observed an increase in the antitumorigenic immune factors IL‐2R and GM‐CSF, which are clinically associated with improved prognoses.^22^, ^23^, ^24^ The expression of the macrophage related CapG protein was suppressed which usually correlates with reduced migration.^25^, ^26^, ^27^ An increase in M‐CSF expression was observed, which is the primary regulator of macrophage survival, proliferation, and differentiation. RANCE‐1 significantly decreased the expression of, HIF‐1a, ICAM, VE‐Cadherin, and vascular cell adhesion molecule‐1 (VCAM‐1). The in vivo efficacy was accompanied by a significant decrease in the prometastatic factors cathepsin B (CtsB), cathepsin S (CtsS), FoxC2, MMP‐2, MMP‐3, MMP‐8, ADAM8, and ADAMP9, all of which are peritumoral proteolytic factors known to drive angiogenesis, migration, invasion, and metastasis.^28^, ^29^, ^30^, ^31^ A decrease in CCL7 and CCL8 levels upon RANCE‐1 treatment was also observed, which could decrease the tumor's ability to recruit monocytes, thus rendering the tumor ecosystem more hostile to tumor progression. CCL8 is expressed by dermal fibroblasts to modulate tumor‐stroma and tumor‐tumor cross‐talk in the initiation of metastasis, while ICAM is mainly produced and modulated by immune cells or in response to immune cell signal which are absent in monolayer monoculture.^32^, ^33^

In the context of potential anticancer therapeutic agents, these results suggest that RANCE‐1 would be a good antimetastatic candidate as it inhibits a series of key proteolytic factors (MMPs, ADAMs, Cts) associated with metastasis whose inhibitions have been reported to curb metastasis. Also, the compound potentiated the expression of immune factors, IL‐2‐R and GM‐CSF, known to be associated with optimal host anti‐cancer immune responses and their presence is correlated with good prognosis.^23^, ^26^ RANCE‐1’s inhibitory capacities toward 5 key growth factors also adds to its therapeutic potential. We have found that it is a single molecule multi‐drug of sorts, as it promotes antitumorigenic immune responses, blocks prometastatic proteolytic factors, and inhibits key growth factors.

### 3.6 Pathology

**Figure 7:**
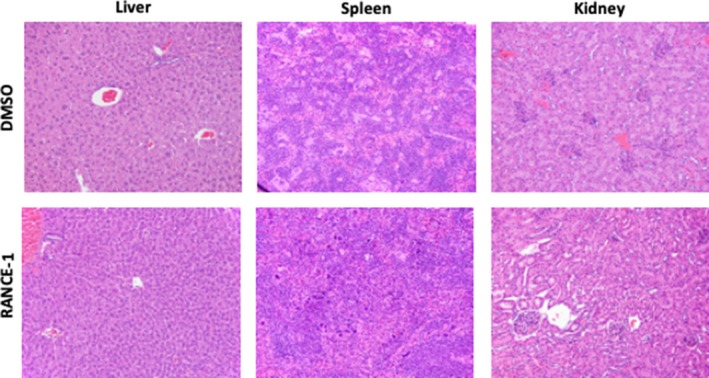
RANCE‐1 treatment does not induce histological changes in liver, spleen, or kidney tissue of mice at the end of the 21 day efficacy trial. Histopathology on H&E staining of paraffin sections magnification 20x. Sections are representative of 3 mice of each treatment

A complete pathology study was performed and revealed that no significant adverse effects were observed after histological evaluation related to the RANCE‐1 treatment (Table 2, Figure 7). Forty‐seven organ and tissue types were analyzed (Heart, Lungs, Thymus, Kidneys, Liver, Gallbladder, Stomach, Duodenum, jejunum, ileum, Cecum, Colon, Mesenteric lymph node, Salivary glands, Submandibular lymph node, Uterus, Cervix, Vagina, Testes/epididymis, Prostate, Seminal vesicles, Urinary bladder, Spleen, Pancreas, Adrenals, Ovaries, Oviducts, Trachea, Esophagus, Thyroid, Parathyroid, Skin (trunk), Mammary glands, Bones (femur, tibia, sternum, vertebrae), Bone marrow (femur, tibia, sternum, vertebrae), Stifle joint, Skeletal muscles (hind limb, spine), Nerves (hind limb, spine), Spinal cord, Oral cavity, Teeth, Nasal cavity, Eyes, Harderian gland, Bones (skull), Pituitary, Brain, Ears, Other organs) and indicated no pathological adverse‐consequences from RANCE‐1, a physicochemical study and complete blood count were performed and no significant changes was observed as a result of treatment. We found no notable difference in total body weight or organ weight between the treated and control mice at the end of the trial, the data also suggest there were no enlargements or atrophy as a result of treatment.

**Table 2:** Summary of histological data

Average weight (g)	RANCE‐1	Vehicle
Body	21.31 ± 5.1	22.82 ± 2.14
Liver	1.15 ± 0.37	1.17 ± 0.28
Spleen	0.04 ± 0.017	0.05 ± 0.01
Heart	0.11 ± 0.05	0.13 ± 0.02
Average Kidney	0.17 ± 0.06	0.16 ± 0.06

Following a 21 day course of treatment with RANCE‐1 or Veh. control once/72h, mice were euthanized, organs were collected via necropsy and weighed immediately after collection. The means and SEM are indicated in the table, n = 3 mice per group.

All kidney health indicators were within normal values. Finally, we found no differences in the clinical chemistry of the treated or control animals (Sup. Table S1), indicating normal production and excretion of physiological fluids and metabolic markers whose deregulation are indicators of pathology or drug side effects.

**Chart 2:**
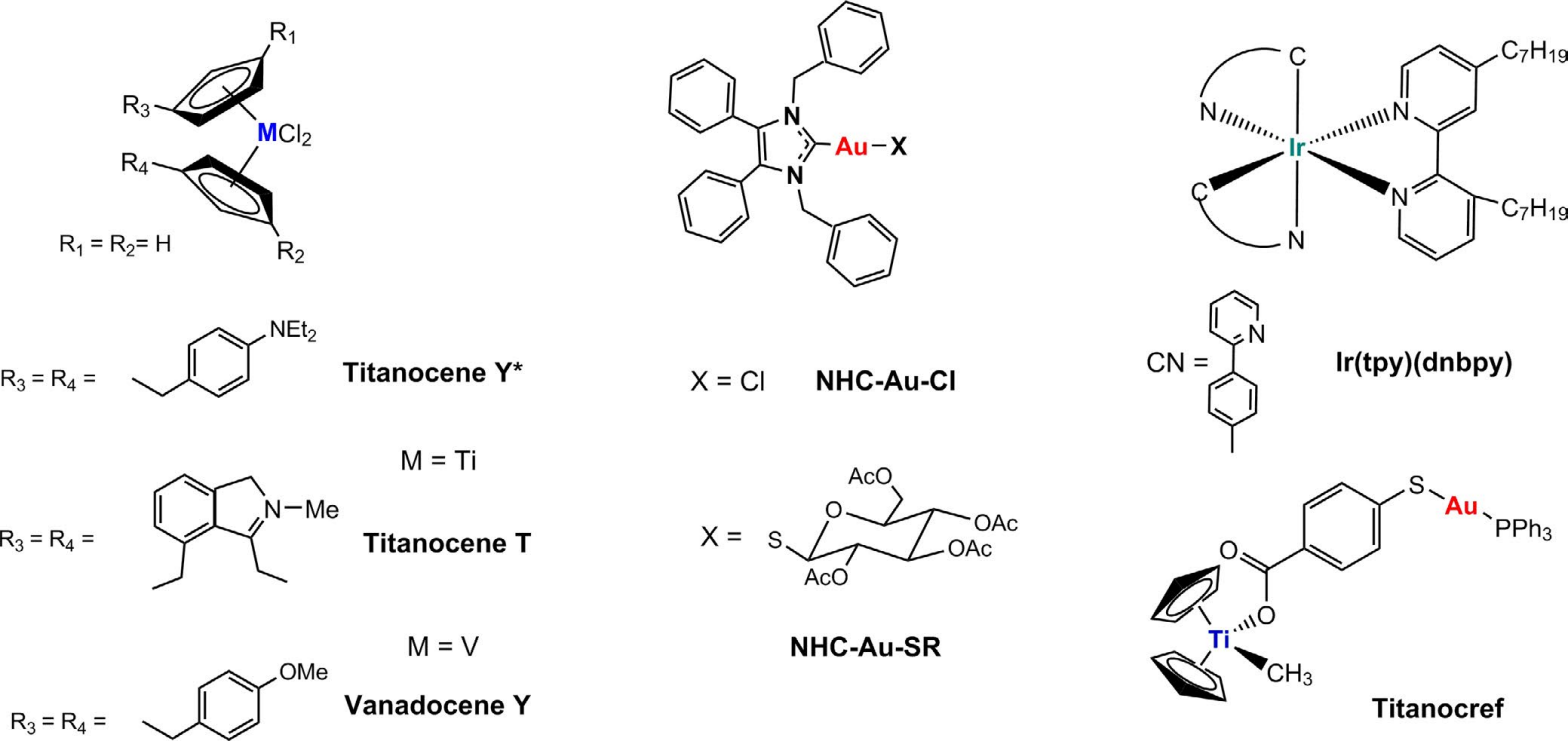
Metal‐based compounds studied in vivo in renal cancer mice xenograft models.^15^, ^34^, ^35^, ^36^, ^37^, ^38^

Other metal‐based drugs (Chart 2) have been tested in vivo in renal cancer mice xenograft models.^16^, ^17^, ^34^, ^35^, ^36^, ^37^, ^38^ Titanium and vanadium compounds such as Titanocene Y*,^34^ Titanocene T^35^ and Vanadocene Y^36^ decreased tumor growth in Caki‐1 tumor‐bearing female NMRI:nu/nu mice (T/C values ranging from 76% to 20%) and no severe side effects were observed. In the case of Titanocene Y*,^34^ the proliferation marker ki‐67 was reduced by 21% and there was decreased vascularization. However this compound caused dose‐dependent weight loss. More recently,^37^ 2 organometallic Au compounds containing N‐heterocyclic carbenes (NHC‐Au‐Cl and NHC‐AuSR) showed a decrease in tumor growth in the same xenograft mice model at T/C 47% with no severe side effects. In these cases, there was no decrease of ki‐67 or CD31 markers.^37^ An organometallic iridium(III) compound ([Ir(tpy)(dnbpy)] showed tumor growth inhibition in A431 tumor‐bearing female BALB/cA.Cg‐Foxn1^nu^/CrINarI while inhibiting H‐Ras/Raf‐1 interaction.^38^ Our group reported on the impressive tumor reduction (67%) after treatment for 28 days in Caki‐1 tumor‐bearing NOD.CB17‐Prkdc SCID/J mice of a bimetallic titanium‐Au compound (Titanocref).^16^, ^17^ For these examples, there was no histopathology studies described and only in one case (Titanocref) there was a PK analysis reported which was not conclusive due to incomplete clearance of the compound in the dosing regimen studied.^16^, ^17^ Our study is the first to show not only complete tumor growth inhibition in a renal cancer mice model, but to include mechanistic, pharmacokinetic, and histopathology studies which demonstrate no or minimal side effects. Histopathology and pharmacokinetic studies with metal‐based compounds other than FDA approved platinum compounds are rare and thus of much interest to the scientific community. Moreover, there is a misconception about the toxicity of metal‐based drugs and studies like the one presented here, reinforce the idea that not all metal‐based drugs behave in the same way or have the same type of mode of action. Unconventional metal‐based anticancer agents with low toxicity (like RANCE‐1) should be further explored as potential cancer chemotherapeutics.

## 4 CONCLUSIONS

Heterobimetallic Ru‐Au complex RANCE‐1 drastically inhibits tumor growth in mice bearing xenografted metastasis‐derived CCRCC tumors without prediction of grave clinical side effects as indicated by the pathology study which highlights that there are no evidence of systemic adverse effect resulting from 21 days of treatment with RANCE‐1. In vivo, the inhibition of tumor growth coincided with a significant decrease in proliferation, and a reduction in the expression of growth factors known to drive malignant tumor progression phenotypes including angiogenesis such as VEGF and hyperproliferation such as, PDGF, FGF, EGFR, and HGRF. Changes in expression levels of factors known to modulate tumor growth through immune recruitment (ie, CCL7, CCL8, ICAM) by resistance to death (ieTrxR, Trx, HIF‐1), and metastasis (ie, MMPs, ADAMs, ILs, Cts) may drive the inhibition observed.

There is a salient distinction between in vivo RANCE‐1 efficacy which is cytotoxic in vitro^19^, ^20^ and cytostatic in vivo*,* and their effect on cancer cell behavior. Cytotoxic drugs act by stimulating cell death by triggering apoptosis, which is the efficacy profile of RANCE‐1^20^ in vitro. Whereas, cytostatic drugs act by inhibiting the hyperproliferation of cancerous cells thus blocking cell growth, which seems to be the in vivo efficacy of RANCE‐1. Since RANCE‐1 seems able to block this proliferation and thus inhibit tumor growth, it holds clinical potential. An added asset of RANCE‐1 in addition to tumor growth inhibition is its favorable pathology profile which would suggest no significant clinical side effects, since all tissue analyzed appear healthy. The efficacy of RANCE‐1 seems to be linked to reduction of activity of protumorigenic growth factors, the modulation of immune cell markers and the inhibition of proteins whose expression is associated with chemoresistance all of those features might make RANCE‐1 a good candidate for combination therapy with cytotoxic agents hindered by chemoresistance (eg, 5FU or Gemcitabine). The inhibition of key growth factors might be a potentiating addition to other growth factor inhibitors creating a pan‐growth‐factor inhibitor cocktail. In the event of inoperable cancers, controlling the growth of is critical, thus an agent such as RANCE‐1 might be a good candidate. It is worth reiterating that the indications from the pathology study shows that RANCE‐1 is likely to cause no or minimal side effects makes it an appealing compound to potentiate drugs whose efficacy is associated with significant adverse effects.

The landscape of CCRCC treatment is terse. Currently available pharmaceutical interventions are associated with limited efficacy since neither cytotoxic chemotherapies, nor targeted or immunotherapies have to date successfully cured advance renal cancer and without significant side effects. Thus, there is merit to clinical interventions that can inhibit malignant progression of solid tumors.

## CONFLICT OF INTEREST

The authors declare no conflict of interest.

## AUTHOR CONTRIBUTIONS

BTE designed and executed the intervention trial, co‐wrote the manuscript and prepared the tables and figures. YP carried out the protein analysis. BL executed the pharmacokinetic experiments and contributed to writing the pharmacokinetic section of the manuscript. SP oversaw the execution of both the pharmacokinetic study and writing of the report of the pharmacokinetic study. M.C and KHP contributed to the design of the study, the interpretation of the results, supervision of the research, and writing of the manuscript.
