# Supplementary material for: Preclinical evaluation of an unconventional ruthenium‐gold‐based chemotherapeutic: RANCE‐1, in clear cell renal cell carcinoma
Source: Cancer Med. 2019 Jun 13;8(9):4304–14. doi: 10.1002/cam4.2322 (PMC6675714; doi:10.1002/cam4.2322)
Supplement: Supplementary file 1 [file CAM4-8-4304-s001.docx]

Original Article

Preclinical Evaluation of an Unconventional Ruthenium-Gold-Based Chemotherapeutic: RANCE-1, in Clear Cell Renal Cell Carcinoma

Benelita T. Elie, PhD1,2; Karen Hubbard, PhD2,3; Yuriy Pechenyy, MS3; Buddhadev Layek, PhD^4^; Swayam Prabha, PhD4; Maria Contel, PhD1,2,5,6

**Corresponding authors:** Maria Contel, PhD, Department of Chemistry, Brooklyn College, The City University of New York, 2900 Bedford Avenue, Brooklyn, NY 11210 (USA), email: [mariacontel@brooklyn.cuny.edu](mailto:mariacontel@brooklyn.cuny.edu); Karen Hubbard, PhD, Department of Biology, City College of New York, The City University of New York, 160 Convent Ave, New York, NY 10031 (USA), email: [khubbard@ccny.cuny.edu](mailto:khubbard@ccny.cuny.edu).

^1^Department of Chemistry, Brooklyn College, The City University of New York; Brooklyn, New York; ^2^Biology, ^5^Chemistry and ^6^Biochemistry PhD Programs, The Graduate Center, The City University of New York, New York, New York; ^3^Department of Biology, City College of New York, The City University of New York, New York, New York; ^4^University of Minnesota. College of Pharmacy, Minneapolis, Minnesota.

**Supporting Information**

# Table of Contents

**1.** Summary of Plasma Chemistry Parameters 2

**2.** Summary of Hematology Values 3

**3.** Summary of Histopathological Findings 4

**Supplementary Table 1**. Summary of plasma chemistry parameters metabolic analytes for three specimens (collected 72h after the last dose of 10mg/kg/72h at the end of a 21 day trial) from 3 tumor bearing NOD-SCID (NOD/LtSz-Prkdscid/j. The study criteria reflect pathological criterion used in clinical studies. Metabolic markers were quantified from freshly isolated plasma and compared to reference range.

|  |  |  | **Reference ranges** |
| --- | --- | --- | --- |
|  | **Veh** | **RANCE-1** |  |
| **BUN (mg/dL)** ***▴** | 26 | 18.33 | 5.0-28 |
| **CREA (mg/dL)** | 0.18 | 0.117 | 0.2-0.5 |
| **BUN/CREA ratio *▴** | 144.4 | 183.33 | - |
| **ALP (U/L) *▾** | 75 | 39.33 | 105-370 |
| **ALT (U/L) *▴** | 92 | 170.66 | 27-195 |
| **AST (U/L)** | 312 | 394.67 | 54-77 |
| **GGT (U/L)** | 0.0 | 0 | - |
| **TBIL (mg/dL)** | 0.2 | 0.23 | 0.2-0.6 |
| **DBIL (mg/dL)** | 0.0 | 0.033 | - |
| **IBIL (mg/dL)** | 0.2 | 0.2 | - |
| **TP (g/dL)** | 5.1 | 5.2 | 4.8-7.2 |
| **ALB (g/dL)** | 2.9 | 2.57 | 2.4-4.3 |
| **GLOB (g/dL)** | 2.2 | 2.63 | 1.7-2.2 |
| **A/G ratio** | 1.3 | 0.99 | - |
| **P (mg/dL)** | 10.7 | 10.37 | 7.3-14.5 |
| **Ca (mg/dL)** | 9.9 | 9.67 | 9.5-12.5 |
| **GLU (mg/dL) *▾** | 191 | 165 | 172-372 |
| **CHOL (mg/dL) *▴** | 77 | 87.67 | 55-169 |
| **TRIG (mg/dL) *▾** | 133 | 116 | 67-289 |
| **CK (U/L) *▾ ◎** | 934 | 368.67 | 428-1609 |
| **TCO2 (mEq/L)** | 20 | 18 | - |
| **Na (mEq/L)** | 153 | 150.67 | 145-181 |
| **K (mEq/L)** | 9.2 | 10.97 | 7.3-11.1 |
| **Cl (mEq/L)** | 113 | 106 | 111-134 |
| **Na/K ratio** | 17 | 13.89 | - |
| **Anion Gap *▴** | 29 | 37.63 | - |

* P < 0.05, values statistically different between Vehicle treated and RANCE-1 treated groups. ▾ RANCE-1 induce a decrease from Veh treated group, ▴ RANCE-1 induce an increase from Veh treated group, deviates significantly from the normal range ◎

ALP, alakaline phosphatase; AST, aspartate aminotransferase; Ca, calcium; Cl, chloride; CPK, creatine phosphokinase; P, phosphorus; K, potassium; Na, sodium; BUN, blood urea nitrogen; Statistical significance (*P <0.05).

**Supplementary Table 2**. Summary of hematology values (mean) for three specimens (collected 72h after the last dose of 10mg/kg/72h at the end of a 21 day trial (NOD/LtSz-Prkdscid/j. Complete blood counts of each group (n = 3 mice per group). Heparinized blood acquired from mice was assessed using the automatic Hematology Analyzer. Blood smears were carried out and fixed with methanol, stained, and observed under the microscope to carry out differential blood white cell counts.

| **Automatic count** |  |  | **NSG MICE Reference ranges** |
| --- | --- | --- | --- |
|  | **Veh.** | **RANCE-1** |  |
| RBC (M/uL) | 9.49 | 10.31 | 7.84-10.84 |
| HGB (g/dL) | 14.9 | 15.8 | 11.8-17.6 |
| HCT (%) | 49 | 52.8 | 44.1-58.3 |
| MCV (fL) | 51.6 | 51.3 | 51.1-58.6 |
| MCH (pg) | 15.7 | 15.37 | 13.7-17.2 |
| MCHC (g/dL) | 30.4 | 29.9 | 25.1-31.3 |
| RDW-SD (fL) | 30.6 | 31.03 | - |
| RDW-CV (%) | 22.9 | 23.5 | 17.3-20.3 |
| RET# (K/uL) *▾ | 437.5 | 192.67 | 294-444 |
| RET (%) *▾ | 4.61 | 2.03 | 2.56-4.56 |
| PLT (K/uL) *▾ | 455 | 1056.67 | 651-2055 |
| PDW (fL) | 7.2 | 7.5 | - |
| MPV (fL) | 6.4 | 6.53 | 4.2-6.3 |
|  |  |  |  |
| **Morphology** | 4+ polychromasia. 2+ Echinocytes. 3+ PLT clumps. | 2+ PLT clumps. RBC morphology is within normal limits. 4+ polychromasia. 2+ acanthocytes. 4+ polychromasia. 2+ acanthocytes. |  |
|  |  |  |  |
| **Manual differential** |  |  |  |
|  | **Veh.** | **RANCE-1** | **NSG MICE Reference ranges** |
| Neut# (K/uL) *▴, ◎ | 1.40 | 8.55 | 0.54-3.16 |
| Band# (K/uL) | 0.00 | 0 | - |
| LYMPH# (K/uL) *▴ | 1.21 | 0.616666667 | 0.23-1.56 |
| MONO# (K/uL) | 0.00 | 0 | 0.03-0.26 |
| EO# (K/uL) | 0.00 | 0.016666667 | 0.00-0.39 |
| NEUT (%) *▴ | 51 | 80.66666667 | 44.21-79.92 |
| LYMPH (%) *▾ | 44 | 15.66666667 | 13.51-42.61 |
| MONO (%) *▾ | 5 | 2 | 1.71-10.93 |
| EO (%) | --- | --- | 0.29-10.32 |
|  |  |  |  |
| **Morphology** | The sample contains a large amount of macrophages. | Many neutrophils are hypersegmented. |  |
|  |  |  |  |

* P < 0.05, values statistically different between Vehicle treated and RANCE-1 treated groups. ▾ RANCE-1 induce a decrease from Veh treated group, ▴ RANCE-1 induce an increase from Veh treated group, deviates significantly from the normal range ◎

WBC: white blood cells, RBC: red blood cells, Hgb: hemoglobin, HCT: hematocrit, MCV: mean cell volume, MCH: mean corpuscular hemoglobin content, MCHC: mean corpuscular hemoglobin concentration, RDW-CV: red cell distribution width-coefficient of variation, PLT: platelets, MPV: mean platelet volume.

**Supplementary Table 3**. Histology. Summary of Histopathological Findings.

|  | **Veh.** | **Rance-1** |
| --- | --- | --- |
| **Body weight (g)** | 21.871 | 18.731 |
| **Liver weight (g)** | 1.112 | 0.974 |
| **Spleen weight (g)** | 0.055 | 0.035 |
| **Heart weight (g)** | 0.107 | 0.078 |
| **Left Kidney weight (g)** | 0.139 | 0.148 |
| **Right Kidney weight (g)** | 0.134 | 0.140 |
| **Heart** | N | N |
| **Lungs** | N | N |
| **Thymus** | N | N |
| **Kidneys** | N | N |
| **Liver** | N | Minimal extramedullary hematopoiesis. Minimal hepatocyte hypertrophy. Mild vacuolar change (microvesicular) |
| **Gallbladder** | N | N |
| **Stomach** | N | N |
| **Duodenum, jejunum, ileum** | N | Mild peritonitis and serositis, neutrophilic and histiocytic, multifocal. |
| **Cecum** | Typhlitis and peritonitis, neutrophilic, with mural edema, multifocal, mild | N |
| **Colon** | N | N |
| **Mesenteric lymph node** | N | N |
| **Salivary glands** | N | N |
| **Submandibular lymph node** | N | N |
| **Uterus** | N | N |
| **Cervix** | N | N |
| **Vagina** | N | N |
| **Testes/epididymis** | N | N |
| **Prostate** | N | N |
| **Seminal vesicles** | N | N |
| **Urinary bladder** | N | N |
| **Spleen** | Mild increased extramedullary hematopoiesis | Mild increase in extramedullary hematopoiesis with granulocyte hyperplasia. |
| **Pancreas** | N | Minimal peritonitis, neutrophilic and histiocytic, multifocal |
| **Adrenals** | N | N |
| **Ovaries** | N | N |
| **Oviducts** | N | N |
| **Trachea** | N | N |
| **Esophagus** | N | N |
| **Thyroid** | N | N |
| **Parathyroid** | N | N |
| **Mammary glands** | N | N |
| **Bones (femur, tibia, sternum, vertebrae)** | N | N |
| **Bone marrow (femur, tibia, sternum, vertebrae)** | N | Moderate diffuse granulocytic hyperplasia; Moderate diffused Erythroid hypoplasia. |
| **Stifle joint** | N | N |
| **Skeletral muscles (hind limb, spine)** | N | N |
| **Nerves (hind limb, spine)** | N | N |
| **Spinal cord** | N | N |
| **Oral cavity** | N | N |
| **Teeth** | N | N |
| **Nasal cavity** | N | N |
| **Eyes** | N | N |
| **Harderian gland** | N | N |
| **Bones (skull)** | N | N |
| **Pituitary** | N | N |
| **Brain** | N | N |
| **Ears** | N | N |
|  |  |  |
| **Legend** |  |  |
| N: Normal |  |  |
